# Supplementary material for: Targeting the Bet-Hedging Strategy with an Inhibitor of Bacterial Efflux Capacity Enhances Antibiotic Efficiency and Ameliorates Bacterial Persistence In Vitro
Source: Microorganisms. 2022 Oct 5;10(10):1966. doi: 10.3390/microorganisms10101966 (PMC9609472; doi:10.3390/microorganisms10101966)
Supplement: Supplementary file 1 [file microorganisms-10-01966-s001.zip › microorganisms-1920707-supplementary-updated.pdf]

microorganisms

Supplemetary materials

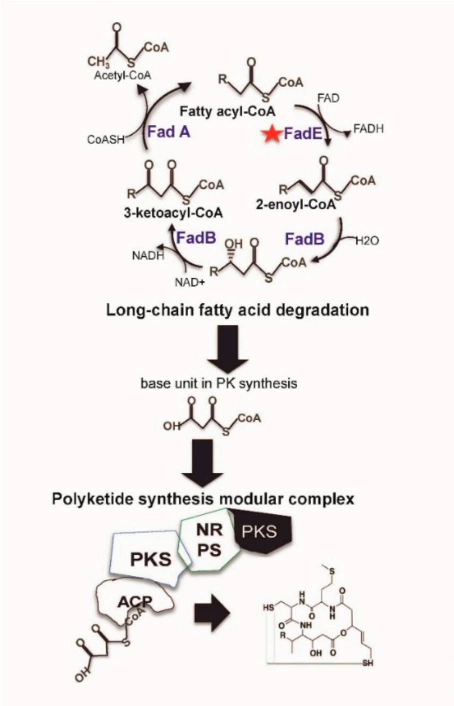

Figure S1. Schematic presentation showing long-chain fatty acids beta-oxidation pathway providing building block molecule to the PKS function. Malonyl-CoA, a product of the long-chain fatty acid degradation serves as a base unit in the polyketide (PK) synthesis [36]. The product of the PKS (BTH_RS24270) function (burkholdac) has been first purified and categorized as inhibitor of mammalian histone deacetylase enzymes [28].
